# Association Between Red Cell Distribution Width and Glycated Hemoglobin in Type 2 Diabetes Mellitus: A Case-Control Study

**DOI:** 10.7759/cureus.106858

**Published:** 2026-04-11

**Authors:** Sarah Afzal, Alen Thomas, Sravanthi Devulapalli, Anjali Khare, VS Bhargav Pradeep Konakanchi, Nishal A Kumar, Shamim U Rahman, Aditya Seth, Srinivasa Rajasekhar Kata, Nagarjuna Sivaraj

**Affiliations:** 1 Medicine, Subharti Medical College, Meerut, IND; 2 Nephrology, Max Super Speciality Hospital, New Delhi, IND; 3 Medicine, FH Medical College, Agra, IND; 4 Research and Development, Great Eastern Medical School and Hospital, Srikakulam, IND; 5 Pathology, Subharti Medical College, Meerut, IND; 6 Internal Medicine, Andhra Medical College, Visakhapatnam, IND; 7 Emergency Medicine, Midland Metropolitan University Hospital, Birmingham, GBR; 8 General Internal Medicine, Andhra Medical College, Visakhapatnam, IND; 9 Medicine, Safdarjung Hospital, Delhi, IND; 10 Medicine, Sant Parmanand Hospital, Delhi, IND; 11 Medicine, Satyabhama Hospital, Delhi, IND; 12 Preventive Medicine, Shri Guru Ram Rai Institute of Medical and Health Sciences, Dehradun, IND; 13 Internal Medicine, Katuri Medical College and Hospital, Dr. N. T. Rama Rao (NTR) University of Health Sciences, Guntur, IND

**Keywords:** glycated hemoglobin, glycemic control, hba1c, india, red cell distribution width, type 2 diabetes mellitus

## Abstract

Background: Type 2 diabetes mellitus (T2DM) is a global public health concern, particularly prevalent in low- and middle-income countries like India. Glycated hemoglobin (HbA1c) is the gold-standard marker for assessing long-term glycemic control. Red cell distribution width (RDW), an index routinely reported in complete blood count, has recently gained interest for its potential association with diabetes-related complications. This study aimed to evaluate the association between RDW and HbA1c levels in patients with T2DM and assess whether RDW could serve as a supplementary marker for glycemic status.

Methods: A hospital-based analytical case-control study was conducted in the Department of Pathology at a tertiary care teaching hospital over a two-month period from June to July 2021. A total of 200 participants were included, comprising 150 patients with confirmed T2DM and 50 nondiabetic controls. All hematological and biochemical measurements were obtained from blood samples collected at a single time point during the study period. Eligible participants were recruited using consecutive sampling during the study period. Diagnosis of T2DM was based on the American Diabetes Association diagnostic criteria. Participants with anemia, hematologic disorders, malignancies, chronic liver or kidney disease, pregnancy, or acute infections were excluded to minimize confounding influences on hematological parameters. Continuous variables were expressed as mean ± standard deviation. Intergroup comparisons were performed using Student’s t-test, and Pearson’s correlation analysis was used to evaluate the association between HbA1c and RDW. Statistical significance was defined as p < 0.05.

Results: A total of 200 participants were analyzed, with a mean age of 58.27 ± 10.43 years. The diabetic cohort demonstrated significantly higher HbA1c levels (8.84% ± 2.33%) than nondiabetic controls (6.37% ± 1.82%, p < 0.001). However, mean RDW values were comparable between the two groups (13.59% ± 1.31% in diabetics vs. 13.64% ± 2.13% in controls; p = 0.808). Within the diabetic group, a statistically significant but weak positive correlation was observed between HbA1c and RDW (r = 0.126, p = 0.024), suggesting that increased variability in red blood cell size may be associated with poorer glycemic control. Additionally, a modest negative correlation between HbA1c and age was identified (r = -0.205, p = 0.012). No significant association between RDW and HbA1c was observed in the nondiabetic control group.

Conclusion: The findings suggest a modest but significant association between RDW and HbA1c in individuals with T2DM. Given its cost-effectiveness and availability, RDW may serve as an adjunct marker in diabetes monitoring, particularly in resource-constrained settings. Further large-scale studies are warranted to validate its clinical utility.

## Introduction

Type 2 diabetes mellitus (T2DM) has emerged as a significant global health concern, with its prevalence increasing rapidly worldwide. According to the International Diabetes Federation Diabetes Atlas 2025, approximately 589 million adults aged 20-79 years, representing 11.1% of the global adult population, are living with diabetes, equivalent to roughly one in nine adults globally. The burden is particularly substantial in developing countries such as India [1]. Diabetes poses a major public health challenge due to its association with various long-term complications, including diabetic retinopathy, nephropathy, neuropathy, and cardiovascular diseases. These complications are closely linked to both the degree and duration of chronic hyperglycemia [2]. Glycated hemoglobin (HbA1c) is a key biomarker that reflects average blood glucose levels over the preceding 8-12 weeks, corresponding to the lifespan of red blood cells (RBCs). As such, HbA1c has become the gold standard for diagnosing and monitoring diabetes [3].

Prolonged hyperglycemia and elevated HbA1c levels are known to induce structural and functional changes in RBCs, which can be reflected in red cell indices, particularly the red cell distribution width (RDW) [4]. RDW is a quantitative measure of the variability in RBC size and is routinely generated by automated hematology analyzers as part of a complete blood count (CBC). It is calculated using the width of the RBC volume distribution curve in relation to the mean corpuscular volume (MCV) [5]. While RDW typically increases in cases of anisocytosis (i.e., a broad range of RBC sizes), it can sometimes be elevated even in a morphologically homogeneous cell population [6]. Traditionally, RDW has been used alongside MCV to differentiate various types of anemia [7]. However, its clinical utility has expanded in recent years, with studies demonstrating its prognostic value in conditions such as heart failure [8,9], chronic kidney disease, obesity, malignancies, and inflammatory disorders [10].

The pathophysiological effects of sustained hyperglycemia on RBCs, including altered membrane integrity, oxidative stress, and impaired deformability, may contribute to variations in RDW. This raises the possibility of an association between HbA1c and RDW in individuals with T2DM. Although both parameters are widely used independently in clinical settings, their combined relevance to glycemic assessment remains underexplored. Given that RDW is an inexpensive, routinely available parameter in CBC reports, investigating its correlation with HbA1c could provide an accessible, cost-effective adjunct for monitoring glycemic control, particularly in low-resource settings such as India [11,12]. Therefore, the present study aims to evaluate the association between RDW and HbA1c in patients with T2DM, with the broader goal of identifying additional tools for risk stratification, complication prevention, and improved disease management. Therefore, we aimed to estimate HbA1c and RDW levels from blood samples of patients diagnosed with T2DM and to analyze the correlation between HbA1c and RDW in these patients.

## Materials and methods

A hospital-based analytical case-control study was conducted in the Department of Pathology at a tertiary care teaching hospital over a two-month period from June to July 2021. A total of 200 participants were included, comprising 150 patients with confirmed T2DM and 50 nondiabetic controls. The unequal group distribution was deliberate and justified. A larger diabetic cohort allowed for greater variability in HbA1c levels and enabled subgroup analyses by age, gender, diabetes duration, and glycemic control. The control group served to establish normative hematological ranges rather than to enable matched comparisons. A 3:1 case-to-control ratio ensured statistical power while remaining practical for recruitment and analysis. All hematological and biochemical measurements were obtained from blood samples collected at a single time point during the study period. Eligible participants were recruited using consecutive sampling, wherein all patients meeting the inclusion criteria during the study period were enrolled. Age was obtained from hospital registration records and verified, where available, using patient identification documents at the time of enrollment. The sample size was determined pragmatically based on feasibility, patient availability during the two-month study period, and the exploratory objective of assessing the association between RDW and HbA1c in patients with T2DM. Although a formal a priori sample size calculation was not performed, the sample size is comparable to that used in similar hospital-based studies evaluating hematological parameters in diabetic populations [3,4]. Consequently, the study may be underpowered to detect small effect sizes, particularly given the weak correlation observed.

Participants with a confirmed diagnosis of T2DM were included in the study. Exclusion criteria encompassed individuals with anemia of any cause, leukemia, current pregnancy, chronic systemic illnesses such as chronic liver disease, chronic kidney disease, and autoimmune or rheumatologic disorders. Additionally, patients with acute or chronic infections (e.g., malaria and tuberculosis) or any form of malignancy were excluded. Exclusion criteria were assessed through review of medical history, clinical examination, and available laboratory investigations documented in hospital records. Venous blood samples were collected under aseptic conditions into ethylenediaminetetraacetic acid vials (lavender-top tubes) for hematological analysis. HbA1c levels were estimated using the Bio-Rad D-10 high-performance liquid chromatography system (Bio-Rad Laboratories, Hercules, CA), while RDW was measured as part of the CBC using the Yumizen H2500 automated hematology analyzer (Horiba, France). All hematological parameters and HbA1c values were obtained from blood samples collected at a single time point during the study period. All sample processing was performed in accordance with the respective manufacturer’s guidelines, and the results were extracted from laboratory records for analysis.

The diagnosis of T2DM in this study was based on the diagnostic criteria (Table 1) recommended by the American Diabetes Association (ADA) Standards of Medical Care in Diabetes (2025) [13]. According to these criteria, an individual is classified as diabetic if any one of the following parameters is met: HbA1c ≥6.5%, fasting blood glucose ≥126 mg/dL, or a two-hour plasma glucose level ≥200 mg/dL during an oral glucose tolerance test. For classification purposes, HbA1c values <5.7% are considered normal, 5.7%-6.4% as indicative of prediabetes or high risk, and values ≥6.5% are diagnostic of diabetes, with confirmation required by repeat testing on a separate occasion. For optimal glycemic control in patients with established diabetes, an HbA1c target of <7% is recommended. RDW represents the coefficient of variation in RBC volume. The normal reference range used was 12.1-14.0 fL. Nondiabetic controls were confirmed based on fasting plasma glucose <100 mg/dL and HbA1c <5.7%, in accordance with ADA diagnostic criteria.

**Table 1:** Type 2 diabetes mellitus was diagnosed according to American Diabetic Association guidelines HbA1c: glycated hemoglobin

Parameter	Normal	Prediabetic	Diabetic
Fasting plasma glucose (mg/dL)	<100 mg/dL	100-125	≥126
Two-hour plasma glucose (mg/dL)	<140 mg/dL	140-199	≥200
HbA1c (%)	<5.7	5.7-6.4	≥6.5

HbA1c and RDW values, along with demographic and clinical data (age, sex, hemoglobin, MCV, mean corpuscular hemoglobin (MCH), mean corpuscular hemoglobin concentration (MCHC), and diabetes duration), were recorded in a structured data collection form. Each entry was verified to minimize data entry errors. Data were analyzed using R Software (version 3.6.3; released on 29 February 2020; R Foundation for Statistical Computing, Vienna, Austria) [14]. Continuous variables were expressed as mean ± standard deviation, while categorical variables were presented as frequencies and percentages. Intergroup comparisons between diabetic and nondiabetic individuals were performed using Student’s t-test for continuous data and Pearson’s chi-square test for categorical data. Pearson’s correlation coefficient was used to assess the relationship between RDW and HbA1c. To explore the influence of various demographic and hematologic variables on RDW, participants were grouped based on the upper limit of the reference range for RDW. Minimum, maximum, and median values were calculated across subgroups. Statistical significance was defined as p < 0.05. Due to the modest sample size and exploratory nature of the study, multivariable regression analysis adjusting for potential confounders such as age and gender was not performed.

All subjects were informed about the study's purpose and procedures, and written informed consent was obtained from each participant. Confidentiality of participant identity and medical data was strictly maintained. Relevant clinical details such as age, sex, duration of diabetes, and associated comorbidities were documented. The study protocol received ethical approval from the Institutional Ethics Committee of the University prior to initiation of the study. No artificial intelligence (AI) or generative AI technologies or tools were used in the preparation, writing, analysis, or generation of any part of this manuscript.

## Results

A total of 200 participants were enrolled in the study, comprising 150 individuals with confirmed T2DM (case group) and 50 nondiabetic, healthy individuals (control group). Among the diabetic group, 110 were male (73.3%) and 40 were female patients (26.7%), whereas the control group included 29 male (58%) and 21 female patients (42%). Overall, male patients constituted the majority of the study population (65.5%), with female patients accounting for 34.5%. The mean age of participants in the diabetic group was 59.83 ± 10.61 years, while that of the nondiabetic group was 53.58 ± 8.35 years. The overall mean age of the study participants was 58.27 ± 10.43 years. Demographic details of study participants are summarized in Table 2.

**Table 2:** Demographic details of study participants (n = 200)

Group	Age (years)	Male (%)	Female (%)
All participants	58.27 ± 10.43	131 (65.5%)	69 (34.5%)
Diabetic patients	59.83 ± 10.61	110 (73.3%)	40 (26.7%)
Nondiabetic controls	53.58 ± 8.35	29 (58%)	21 (42%)

HbA1c levels were significantly elevated in the diabetic group (8.84% ± 2.33%) compared to the nondiabetic group (6.37% ± 1.82%, p < 0.001). No statistically significant differences were observed between the two red cell distribution width - coefficient of variation (RDWcv) groups with respect to hemoglobin, MCV, MCH, MCHC, or duration of diabetes (p > 0.05 for all). However, significant differences were noted in age (p = 0.037), gender distribution (p = 0.001), HbA1c levels (p = 0.026), and hematocrit (HCT) (p = 0.0002). The mean RDW was 13.59 ± 1.31 in the diabetic and 13.65 ± 2.14 in the nondiabetic groups (p = 0.808) (Table 3). Among diabetic patients, HbA1c levels were highest in those aged ≥70 years (8.95% ± 2.35%) and lowest in those under 50 years (8.81% ± 2.35%). No clear trend or significant difference was observed in RDW across age categories (Table 4). Male and female diabetic patients had comparable RDW values (13.59 ± 1.31), though female patients exhibited slightly higher HbA1c levels (8.85% ± 2.31%) than male patients (8.78% ± 2.30%). These differences were not statistically significant. When stratified by duration of diabetes (<5 years, 5-10 years, >10 years), neither HbA1c nor RDW showed significant variation (Table 4). Additionally, no significant difference in RDW was found between diabetic patients with good glycemic control (HbA1c <7%) and those with poor control (HbA1c ≥7%).

**Table 3:** Comparison of hematological parameters in controls and cases (n = 200) HbA1c: glycated hemoglobin; HGB: hemoglobin concentration in blood; HCT: hematocrit; MCV: mean corpuscular volume; MCH: mean corpuscular hemoglobin; MCHC: mean corpuscular hemoglobin concentration; RDW-CV: red cell distribution width - coefficient of variation

Parameter	All patients	Nondiabetic (n = 50)	Diabetic (n = 150)	p value	Reference range
HbA1c (%)	8.22 ± 2.47	6.37 ± 1.82	8.84 ± 2.33	0.000	<5.7% (normal); 5.7%-6.4% (prediabetes); ≥6.5% (diabetes)
HGB (g/dL)	12.77 ± 2.07	13.90 ± 2.31	12.64 ± 1.97	0.135	Male: 13-17; female: 12-15
HCT (%)	39.01 ± 5.93	40.35 ± 6.38	38.56 ± 5.70	0.086	Male: 40-54; female: 36-48
MCV (fL)	89.11 ± 8.13	89.46 ± 9.48	89.00 ± 7.61	0.750	80-100
MCH (pg)	29.17 ± 2.95	29.16 ± 3.35	29.17 ± 2.80	0.932	27-33
MCHC (g/dL)	32.71 ± 1.05	32.61 ± 0.97	32.74 ± 1.08	0.384	32-36
RDWcv (%)	13.60 ± 1.55	13.64 ± 2.13	13.59 ± 1.31	0.808	11.5-14.5

**Table 4:** Mean HbA1c and RDW by subgroups in diabetic patients (n = 200) RDW: red cell distribution width; HbA1c: glycated hemoglobin

Subgroup	Mean HbA1c (%)	Mean RDW (%)	p value
Age			
<50 years	8.81 ± 2.35	13.59 ± 1.31	0.133
50-60 years	8.91 ± 2.35	13.56 ± 1.33	0.128
60-70 years	8.84 ± 2.34	13.59 ± 1.31	0.147
≥70 years	8.95 ± 2.35	13.58 ± 1.31	0.146
Gender			
Male	8.78 ± 2.30	13.59 ± 1.31	0.145
Female	8.85 ± 2.31	13.59 ± 1.33	0.142
Duration of diabetes			
<5 years	8.77 ± 2.30	13.59 ± 1.31	0.138
5-10 years	8.80 ± 2.29	13.59 ± 1.31	0.152
>10 years	8.85 ± 2.32	13.59 ± 1.35	0.156

**Figure 1:**
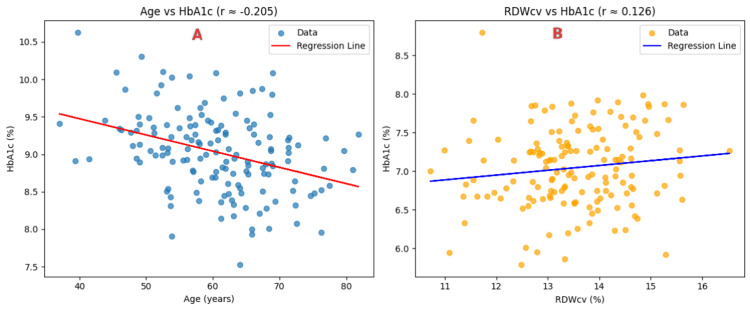
Scatter plots depicting the correlation of HbA1c with age and RDW in the diabetic group (n = 150) (A) Scatter plot showing the correlation between HbA1c and age in diabetic patients. A significant negative correlation was observed (r = -0.205, p = 0.012). (B) Scatter plot showing the correlation between HbA1c and RDW in diabetic patients. A weak but statistically significant positive correlation was observed (r = 0.126, p = 0.024). Pearson’s correlation coefficient was used to assess the associations HbA1c: glycated hemoglobin; RDW: red cell distribution width; RDWcv: red cell distribution width - coefficient of variation

Diabetic patients were further categorized based on RDW values (≤14.0% and >14.0%) (Table 5). Patients with RDW >14.0% were older (median age: 60 vs. 57 years, p = 0.037) and had a higher proportion of female patients (44.7% vs. 18.4%, p = 0.001). Interestingly, HbA1c levels were significantly lower in the >14.0% RDW group (7.5% vs. 8.3%, p = 0.026). HCT was also significantly lower in this group (p = 0.0002). No significant differences were found in hemoglobin, MCV, MCH, MCHC, or diabetes duration (Table 5). In the nondiabetic control group, HbA1c demonstrated a statistically significant inverse correlation with MCV (r = -0.11, p = 0.047) and MCH (r = -0.08, p = 0.048), suggesting that higher HbA1c levels were weakly associated with smaller and less hemoglobinized RBCs. No other hematological parameters, including RDW, hemoglobin, HCT, or age, showed any significant correlation with HbA1c in the control group. In the diabetic group, a significant negative correlation was observed between HbA1c and age (r = -0.205, p = 0.012), indicating that HbA1c levels tended to decrease with advancing age among patients with T2DM. A positive correlation was also found between HbA1c and RDW (r = 0.126, p = 0.024), implying that greater variability in RBC size may be associated with poorer glycemic control (Figure 1). No statistically significant correlations were found between HbA1c and other hematological indices in the diabetic cohort.

**Table 5:** Comparison of demographic and hematological parameters according to RDW subgroups in diabetic patients (n = 150) RDW: red cell distribution width; HbA1c: glycated hemoglobin; MCV: mean corpuscular volume; MCH: mean corpuscular hemoglobin; MCHC: mean corpuscular hemoglobin concentration

Parameter	RDW ≤ 14.0% (n = 103)	RDW > 14.0% (n = 47)	p value
n (%)	103 (68.67%)	47 (31.33%)	-
Age (years)	57 (40-87)	60 41-87)	0.037^*^
Male (%)	84 (81.6%)	26 (55.3%)	0.001^*^
Female (%)	19 (18.4%)	21 (44.7%)	
HbA1c (%)	8.3 (4.3-15.8)	7.5 (4.3-14.0)	0.026^*^
Hemoglobin (g/dL)	12.7 (6.0-17.1)	11.7 (7.2-15.3)	1.043
Hematocrit (%)	38.6 (18.5-52.1)	36.1 (21.3-47.7)	0.0002^*^
MCV (fL)	89.0 (59.2-114.0)	85.0 (59.2-99.0)	0.795
MCH (pg)	29.1 (19.2-37.1)	27.5 (19.2-32.3)	0.136
MCHC (g/dL)	32.7 (30.0-35.6)	32.1 (29.0-34.7)	0.260
Duration of diabetes (years)	6 (1-21)	6 (1-20)	0.410

## Discussion

T2DM is characterized by chronic hyperglycemia resulting from insulin resistance and β-cell dysfunction, which leads to widespread metabolic and vascular disturbances. One of the less explored but clinically significant consequences of persistent hyperglycemia is its impact on RBC morphology and turnover. HbA1c, a marker of long-term glycemic control, reflects the extent of glucose exposure over the RBC lifespan. Prolonged hyperglycemia can induce oxidative stress and low-grade inflammation, which may impair erythropoiesis, reduce RBC survival, and alter membrane deformability. These changes contribute to anisocytosis, a hallmark of elevated RDW [15,16]. RDW, routinely measured as part of the CBC, may reflect underlying hematologic and metabolic alterations in T2DM; however, its role as a surrogate marker requires further validation. Multiple studies across diverse populations have reported a positive association between RDW and HbA1c, suggesting that RDW may reflect underlying hematological alterations associated with chronic hyperglycemia in individuals with T2DM [4,15-17]. While these findings indicate a potential relationship, the evidence remains insufficient to support integrating RDW into comprehensive diabetes risk assessment or diagnostic protocols without further validation through large-scale prospective studies.

Interestingly, findings from a study by Engström et al. conducted in a Swedish population reported an inverse relationship, where lower RDW values were associated with a higher risk of developing T2DM [4]. This divergence in results across populations highlights the need to further explore the relationship between RDW and glycemic markers in region-specific cohorts. In the present study, HbA1c levels showed modest variation across age groups, with slightly higher mean values observed among participants aged ≥70 years (8.66%). Although this may suggest relatively poorer glycemic control in older individuals, the differences across age categories were not statistically significant. Therefore, the findings should be interpreted with caution. Additionally, a weak but statistically significant negative correlation between age and HbA1c was observed in the diabetic cohort, indicating that HbA1c levels tended to decrease slightly with advancing age. These findings may reflect age-related differences in treatment patterns, disease duration, or metabolic regulation among patients with T2DM. Nevertheless, epidemiological studies have consistently demonstrated that the prevalence of diabetes increases with age, with incidence rates rising substantially among individuals older than 60 years [18]. Further, age-related physiological changes, polypharmacy, comorbidities, and diminished glucose tolerance may all contribute to this trend, thereby increasing the risk of acute metabolic derangements and long-term complications in older adults [18,19].

A key finding of this study was the statistically significant weak positive correlation between HbA1c and RDWcv in diabetic patients (r = 0.126, p = 0.024). No such association was observed in the nondiabetic control group. Although statistically significant, the correlation between HbA1c and RDW was relatively weak, suggesting that findings should be interpreted with caution. This suggests that among individuals with T2DM, higher RDW may reflect poorer glycemic control. Also, age and gender are known to influence hematological parameters, including RDW. In the present study, significant differences in age and gender distribution were observed between the groups. As no multivariable adjustment was performed, the observed association between HbA1c and RDW may be influenced by residual confounding. The underlying mechanism for this association could involve hyperglycemia-induced oxidative stress and glycation, which compromise RBC membrane integrity and increase osmotic fragility [20]. This may lead to a reduction in RBC lifespan and compensatory release of newly formed erythrocytes into circulation, creating a more heterogeneous RBC population and thereby elevating RDW. However, this effect may plateau beyond a certain HbA1c threshold (e.g., ≥14.0%), as previously suggested by Engström et al. [4]. This indicates that the relationship between RDW and hyperglycemia may be nonlinear and subject to biological limits in red cell adaptation. Overall, these findings are consistent with the hypothesis that hyperglycemia may influence red cell morphology and turnover, as reflected in red cell indices such as RDW. Therefore, RDW may be considered an additional parameter for evaluating glycemic status, although its clinical utility remains limited and requires further validation.

This study has several limitations that should be considered while interpreting the findings. First, the sample size was relatively modest and drawn from a single tertiary care center, using consecutive sampling, which may limit the generalizability of the results to broader populations. Additionally, no formal a priori sample size calculation was performed, and the sample size was determined pragmatically based on feasibility and participant availability during the study period. Furthermore, the absence of a formal sample size calculation limits the ability to assess statistical power, increasing the risk of type II error and potentially affecting the precision and reliability of the observed associations. The case-to-control ratio was skewed toward the diabetic group to allow for subgroup analyses; however, this imbalance, along with the use of consecutive (nonprobability) sampling, may introduce selection bias and limit external validity. Furthermore, there were notable differences in age and gender distribution between the diabetic and control groups, both of which may act as potential confounding factors influencing hematological parameters such as RDW. Due to the exploratory nature of the study and sample size constraints, no multivariable adjustment or stratified analysis was performed to account for these variables. Importantly, no statistical adjustment for age and gender was undertaken, and therefore, the observed association between RDW and HbA1c may be influenced by residual confounding. Additionally, the cross-sectional and observational nature of the study, with all measurements obtained at a single time point, precludes any inference of causality between RDW and glycemic control. Other potential confounding factors, such as nutritional status, iron levels, inflammatory conditions, or undiagnosed subclinical disorders that may influence RBC indices, were not systematically assessed. Future research should focus on larger, multicenter studies with more balanced group characteristics, formal sample size estimation, and appropriate statistical adjustment, as well as longitudinal designs, to validate and expand upon these findings.

## Conclusions

This study demonstrated a statistically significant but weak positive correlation between HbA1c and RDW in individuals with T2DM, indicating a possible association between RDW and glycemic status. Given the small effect size and potential influence of unadjusted confounding factors, RDW should be interpreted cautiously and cannot be considered a reliable marker for glycemic control based on the present findings. The findings should be interpreted with caution, as the observed association may be influenced by confounding factors such as age and gender. While RDW is an inexpensive and routinely available component of the CBC, its clinical applicability in diabetes assessment remains limited. Further large-scale, adequately powered, multicenter studies with balanced populations and appropriate statistical adjustment are required to validate this association and determine its potential clinical relevance.
